# Platelet-activating factor enhancement of calcium influx and interleukin-6 expression, but not production, in human microglia

**DOI:** 10.1186/1742-2094-2-11

**Published:** 2005-04-15

**Authors:** Prasongchai Sattayaprasert, Hyun B Choi, Sukumal Chongthammakun, James G McLarnon

**Affiliations:** 1 Department of Pharmacology and Therapeutics, Faculty of Medicine, University of British Columbia, Vancouver, BC, Canada; 2 Division of Neurology, Department of Medicine, University of British Columbia, Canada; 3 Department of Anatomy, Mahidol University, Bangkok, Thailand

**Keywords:** Microglia, platelet-activating factor, interleukin-6, store-operated channels

## Abstract

Calcium-sensitive fluorescence microscopy and molecular biology analysis have been used to study the effects of platelet-activating factor (PAF) on intracellular calcium [Ca^2+^]_i _and IL-6 expression in human microglia. PAF (applied acutely at 100 nM) elicited a biphasic response in [Ca^2+^]_i _consisting of an initial rapid increase of [Ca^2+^]_i _due to release from internal stores, followed by a sustained influx. The latter phase of the [Ca^2+^]_i _increase was blocked by SKF96365, a non-selective store-operated channel (SOC) inhibitor. RT-PCR analysis showed PAF treatment of microglia induced expression of the pro-inflammatory cytokine IL-6 in a time-dependent manner which was blocked in the presence of SKF96365. However, ELISA assay showed no production of IL-6 was elicited at any time point (1–24 h) for microglial exposures to PAF. These findings suggest that PAF stimulation of human microglia induces expression, but not production, of IL-6 and that SOC-mediated [Ca^2+^]_i _influx contributes to the enhanced expression of the cytokine.

## Background

Microglia are resident, immunocompetent cells in the brain. They show functional plasticity and can be activated by a diversity of inflammatory stimuli including ones associated with neurodegenerative diseases [9,18]. The functional responses of microglia following activation include proliferation, phagocytosis and secretion. In the latter case microglia can secrete pro- and anti-inflammatory cytokines, chemokines, neurotrophic factors and excitotoxins such as glutamate [20].

One important inflammatory agent is platelet-activating factor (PAF), an alkyl ether phospholipid compound, which both stimulates and is produced by microglia [13]. PAF contributes to inflammatory responses in the brain and is reported to be upregulated in CNS pathophysiology [2,17]. Acute application of PAF to human microglia induces a biphasic change in levels of intracellular Ca^2+ ^([Ca^2+^]_i_) with an initial rapid phase due to intracellular release from endoplasmic reticulum (ER) stores and a secondary phase due to influx through store operated channels (SOC) [15,31]. Importantly, SOC has been shown to exhibit sustained activation following stimulation of human [31] and rodent [29] microglia. Prolonged entry of Ca^2+ ^through SOC in stimulated microglia could constitute a coupling signal between an activating stimulus and cellular functional response. Indeed, the involvement of sustained Ca^2+ ^responses has been reported as a factor in the production of arachidonic acid by rat microglia [23].

The pro-inflammatory cytokine IL-6 is released from activated microglia and mediates inflammatory responses in brain. Levels of IL-6 in serum and cerebrospinal fluid have been found to be elevated in stroke patients [8,28] and the cytokine has also been implicated in the etiopathology of neurodegenerative disorders such as Alzheimer's disease (AD), Parkinson's disease (PD) and HIV encephalopathy [3,14,25]. Interestingly, some evidence is also available suggesting that under some conditions elevated levels of IL-6 in brain may actually be beneficial [27].

In this study we have examined a role for SOC mediated [Ca^2+^]_i _influx in mediating actions of the inflammatory stimulus PAF to induce IL-6 in human microglia.

## Materials and methods

### Preparation of cells

The procedures for the isolation of human microglia have been previously reported [24]. In brief, human embryonic brain tissues were dissected into small blocks, incubated in phosphate-buffered saline (PBS) containing 0.25% trypsin and 40 μg/ml DNase and then dissociated into single cells by repeated pipetting. Cells were plated in T75 flasks in a medium consisting of Dulbecco's modified Eagle's medium (DMEM) containing 5% horse serum, 5 mg/ml glucose, 25 μg/ml gentamicin, and 2.5 μg/ml amphotericin B. Freely floating microglia were harvested from a medium of mixed cell cultures after 7–10 days of growth in culture flasks and plated on aclar coverslips for identification, on poly-L-lysine-coated glass coverslips for calcium spectrofluorometry and plated on six-well multiplates for RT-PCR or ELISA. CD11b and ricinus communis agglutinin (RCA), specific markers for microglia, were used to confirm purity of the culture which was in excess of 98% [24,30].

### Calcium spectrofluorometry

The procedures used for measurement of intracellular Ca^2+ ^have been reported [6,31]. Microglia were incubated with 1 μM fura-2/AM (acetoxymethyl ester, Molecular Probes, Eugene, OR) plus 1 μM pluronic acid in normal physiological saline solution (PSS) for 30 min. PSS solution contained (in mM): NaCl (126), KCl (5), MgCl_2 _(1.2), HEPES (10), D-glucose (10) and CaCl_2 _(1); pH of 7.4. All reagents were obtained from Sigma (St. Louis, MO).

Following a 20 min wash in dye-free solution, coverslips were placed on the stage of a Zeiss Axiovert inverted microscope employing a ×40 quartz objective lens. Cells were exposed to alternating wavelengths of 340/380 nm at 6 s intervals and emission light passed through a 510 nm filter. An imaging system (Empix Imaging, Mississauga, ON) was used to record fluorescence ratios using a CCD camera (Retiga 1300i, Burnaby, BC). Fluorescence ratios were determined and converted to values of [Ca^2+^]i using published procedures [11]. All experiments were done at room temperature (20–22°C).

### Reverse transcription-PCR and ELISA assay

IL-6 expression was detected with the reverse-transcriptase polymerase chain reaction (RT-PCR). Isolation of RNAs was performed using TRIzol (Gibco-BRL, Gaithersburg, MD, USA) and DNA contamination was eliminated using DNase. cDNA synthesis was done using M-MLV reverse transcriptase (Gibco-BRL). The sequences for the human specific primers for IL-6 as follows: sense primer: 5'-GTGTGAAAGCAGCAAAGAGGC-3'; antisense primer: 5'-CTGGAGGTACTCTAGGTATAC-3'. Human-specific IL-6 signals were generated with the GeneAmp thermal cycler and Amplitaq Gold DNA polymerase (Applied Biosystems, Foster City, CA). The conditions for PCR were as follows: initial denaturation at 95°C for 6 min followed by 28 cycles of denaturation at 95°C for 45 sec, annealing at 56°C for 1 min and extension at 72°C for 1 min. A final extension step at 72°C for 10 min was carried out. PCR products (159 bp) were identified using 1.5% agarose gels containing ethidium bromide and visualized under UV light. GAPDH was used as a reaction standard and human specific primer sequences were as follows: sense primer: 5'-CCATGTTCGTCATGGGTGTGAACCA-3'; antisense primer: 5'-GCCAGTAGAGGCAGGGATGATGTTC-3'. The intensities of each band were measured using NIH image J 1.24 software (National Institutes of Health, Bethesda, MD). Relative mRNA levels for each treatment were normalized to GAPDH.

Enzyme-linked immunosorbent assays (ELISA) were performed according to manufacturer instructions (R & D systems, Minneapolis, MN). Cells were plated on multi-well plates (≈10^5 ^cells/well) and treated with PAF (100 nM) in the absence or presence of SKF96365 (20 μM for 8 hr). The cell-free supernatants were used for analysis of IL-6 production (kit detects IL-6 as low as 0.7 pg/ml). Values were expressed as means ± SEM and statistical significance (*p *< 0.05) was determined using one-way ANOVA and Newman-Keuls multiple comparison post-test.

## Results

### Effects of SKF96365 on SOC-mediated [Ca^2+^]_i _influx by PAF

PAF-induced changes in [Ca^2+^]i from human microglia have previously been reported [15,21,31]. Initial study showed a transient increase in SOC [31] but more recent work has shown PAF application to evoke a sustained phase of SOC following an initial component due to depletion of Ca^2+ ^from intracellular stores [15,21]. The differences in PAF responses is considered in the Discussion.

**Figure 1:**
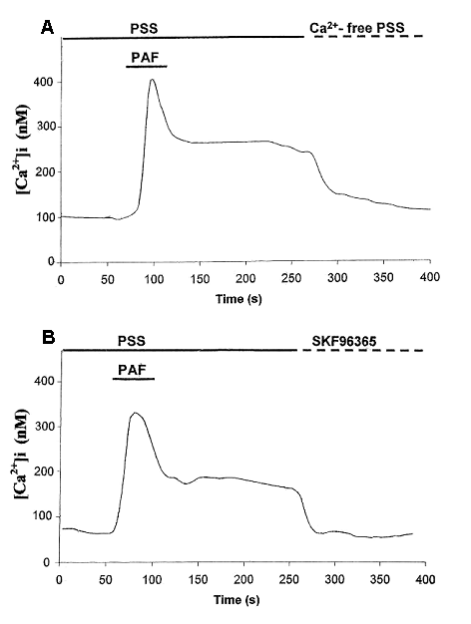
**PAF-induced Ca^2+ ^responses. **A: Representative trace (n = 18 cells) showing change in [Ca^2+^]i induced by PAF (100 nM). Following a prolonged level of SOC-mediated influx of Ca^2+^, the perfusion of Ca^2+^-free PSS abolished the response. B: Results from a separate experiment showing effects of SKF96365 (20 μM) on a PAF-induced increase in [Ca^2+^]i (n = 21 cells). SKF96365 application, during a sustained entry of Ca^2+ ^through SOC, effectively reduced [Ca^2+^] to baseline levels.

A representative response to acute application of PAF (applied at 100 nM) is presented in Fig 1A (n = 18 cells). A plateau level of [Ca^2+^]i was sustained for a duration exceeding 2 min after removal of PAF. Following establishment of a clearly defined plateau phase, the bath solution was replaced with Ca^2+^-free PSS. This procedure caused an immediate decline in [Ca^2+^]i to baseline levels (Fig 1A). Long durations of SOC-mediated influx of Ca^2+ ^have also been documented in mouse microglial cells [29].

The results of application of the SOC inhibitor SKF96365 (at 20 μM) to the plateau phase of a PAF response is shown in the representative recording of Fig 1B (n = 21 cells). SOC-mediated entry of Ca^2+ ^was reduced to baseline values by SKF96365. Amplitude of Ca^2+ ^influx through SOC was measured as the difference between baseline and plateau levels and in five independent experiments (n = 107 cells) the amplitude prior to SKF96365 was 140 ± 21 nM and after SKF96365 was at baseline levels. Previous work has shown SKF96365 pretreatment of human microglia (50 μM for 5 min) abolished a transient SOC in the cells [31].

### Effects of SKF96365 on microglial expression of IL-6

**Figure 2:**
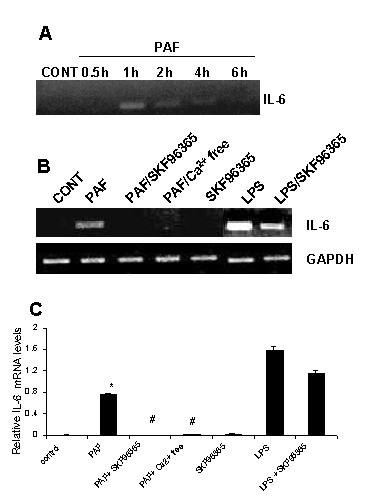
**Expression of IL-6 in PAF treated human microglia. **A: RT-PCR analysis for different exposure times of microglia to PAF (applied at 100 nM). B: Effects of PAF, PAF plus SKF96365, PAF plus Ca^2+^-free and SKF96365 applied alone (1 h treatments). Also shown are effects of LPS and LPS plus SKF96365 (6 hr treatments). GAPDH was used as a reaction standard. C: Semi-quantitative RT-PCR for effects of the different treatments. * *P *< 0.05 compared with unstimulated control; # *P *< 0.05 compared with PAF treated microglia.

We next examined effects of PAF on expression of the pro-inflammatory cytokine IL-6 in the absence and presence of SOC inhibition. The time-dependence of PAF stimulation (100 nM) of human microglia on IL-6 are presented in Fig 2A. The representative RT-PCR showed no constitutive expression of IL-6 in unstimulated microglia (lane 1 of Fig 2A). IL-6 was maximally expressed at 1 h of exposure to PAF then declined to lower levels at longer treatment times (longest exposure of 6 h). A similar time-dependence for IL-6 expression was exhibited in a total of four experiments.

A one hour exposure of human microglia to PAF was chosen for subsequent RT-PCR analysis. As shown in Fig 2B, constitutive expression of IL-6 was absent (lane 1). PAF treatment was effective in stimulating expression of the cytokine (Fig 2B, lane 2). The expression of IL-6 was abolished when SKF96365 was included with the PAF application (Fig 2B, lane 3). No evident IL-6 expression was observed for PAF application in Ca^2+^-free PSS (Fig 2B, lane 4). SKF96365, applied alone in PSS solution, did not cause any increase in IL-6 (Fig 2B, lane 5).

It was of interest to compare PAF as an inducer of microglial IL-6 to that of LPS (lipopolysaccharide) a potent inflammatory stimulus of cells. The results of exposure of human microglia to LPS (100 ng/ml for 6 h) is presented in Fig 2B (lane 6) showing LPS stimulation caused an intense band for IL-6. Altering the number of PCR cycles had no apparent effect on intensity (data not shown) suggesting IL-6 band saturation with LPS (Fig 2B, lane 6). Comparison of band intensity indicated LPS was a more effective inducer of IL-6 relative to PAF. Interestingly, a partial inhibition of LPS-induced IL-6 mRNA was observed when SKF96365 was applied with LPS (Fig 2B, lane 7).

Semi-quantitative RT-PCR analysis is presented in Fig 2C and shows PAF as an effective stimulator of IL-6 expression (n = 3). However, expression of IL-6 was considerably lower with PAF as a stimulus compared with LPS (Fig 2B,C). Inclusion of SKF96365 with PAF or application of PAF in Ca^2+^-free PSS eliminated expression of IL-6 (n = 3). Although LPS was not the subject of this study, the decrease in LPS induction of IL-6 with SKF96365 is of interest and is discussed below.

### ELISA assay for effects of PAF on microglial production of IL-6

**Figure 3:**
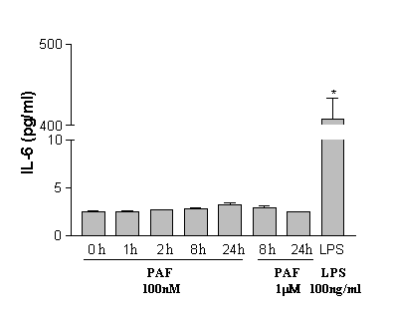
**ELISA assays for production of IL-6 in human microglia. **PAF (at 100 nM) induced no significant production of IL-6 from microglia following exposures from 1–24 h (n = 4 for each time points). PAF (at 1 μM) induced no significant production of IL-6 (following exposures for 8 h and 24 h; n = 3 for both time points); these values are near the lower limits for sensitivity of the ELISA kits. LPS was used as a positive control in these experiments (n = 4); note the change of scale for the ordinate (from 10 to 400 pg/ml). * *P *< 0.05 compared with unstimulated control.

We next investigated production of IL-6 from PAF-treated human microglia using an exposure time of 8 h. No production of IL-6 was evident in four experiments (data not shown); levels of IL-6 were below the detection levels for ELISA assay (≤ 1 pg/ml). In order to determine if the treatment time was a limiting factor in IL-6 production, a series of experiments using different microglial times of exposure to PAF were undertaken (from 1–24 h). The results are presented in Fig 3; no significant production of IL-6 (n = 4) was found for any treatment time (PAF applied for 1,2,8 or 24 h).

We also examined if a ten-fold increase in PAF concentration (to 1 μM) would be effective in producing IL-6. As shown in Fig 3, this higher concentration of PAF also had no effect to induce IL-6 production for treatment times of 8 or 24 h (n = 3 independent experiments). The effects of LPS stimulation were also determined in these experiments (using 100 ng/ml for 8 h). Microglia, treated with LPS, produced high concentrations of IL-6 to levels exceeding 400 pg/ml (n = 4 independent experiments).

## Discussion

The results from this work indicate that PAF-mediated changes in [Ca^2+^]_i _are involved in the cellular expression of the pro-inflammatory agent, IL-6 in human microglia. In essence, activation of SOC acts as a transcriptional control for expression of IL-6. Our results show that inhibition of SOC with SKF96365 blocked both the influx of Ca^2+ ^and microglial expression of IL-6. However, PAF-induced expression of IL-6 (Fig 2) did not translate into production of the cytokine (Fig 3). This result could suggest that an additional signal or factor may be required for microglial secretion of IL-6.

As found for other types of unexcitable cells, microglia do not normally express voltage-dependent Ca^2+ ^channels [7]. The sustained entry of Ca^2+ ^through SOC is likely an important pathway for microglial responses to specific inflammatory stimuli [15,22,26]. Although opening of SOC is required for re-filling of ER stores, other roles for this influx pathway have not been well established. Activation of SOC is necessary for expression of IL-6 but an additional signal is required to produce the pro-inflammatory cytokine in human microglia. The activation state of human microglia may influence the extent of Ca^2+ ^influx through SOC. Microglia showing an ameboid morphology are considered representative of an activated state whereas cells with a ramified morphology are considered quiescent. We have found sustained SOC responses from PAF-stimulated microglia in cells demonstrating ameboid morphology [15,21] and also in the present work. However, an initial study using a mixture of ameboid and ramified shaped cells, showed a transient SOC response with stimulation by PAF [31]. Further work will be useful to correlate expression of SOC with cell activation.

A recent review has provided a detailed overview of ATP as an inducer of IL-6 expression and production in MG-5 microglial cell line [12]. ATP and the purinergic agonist BzATP were both effective in increasing expression of IL-6 with effects involving activation of the p38 MAPK pathway. However, ATP (activator of both metabotropic P2YR and ionotropic P2XR) but not BzATP (activator of the ionotropic subtype P2X_7_R), was found to induce production of the cytokine. The role of SOC in MG-5 cell responses is unclear since ATP evokes a monophasic change in [Ca^2+^]i due to P2YR dependent release from intracellular stores. In human microglia we have attributed the lack of a SOC phase of [Ca^2+^]i due to concomitant ATP binding to some P2XR (not P2X_7_R) causing cellular depolarization and block of Ca^2+ ^influx [6].

PAF induction of IL-6 was found to be time-dependent (Fig 2A) in addition to the dependence on the presence of extracellular Ca^2+ ^and SOC (Fig 2B). We observed no IL-6 expression at one-half hour and a maximal level at one hour of microglial exposure to PAF. Little or no IL-6 was expressed with longer PAF treatments of microglia. Inhibition of endoplasmic reticulum Ca^2+ ^ATPase (SERCA) has been reported to increase IL-6 mRNA expression in rodent macrophages within 15 min [4,19]. Blockade of SERCA, by compounds such as thapsigargin, and subsequent depletion of intracellular stores is a stimulatory protocol for activation of SOC. However, SOC-mediated entry of Ca^2+ ^was not determined in the rodent studies.

Although PAF was an effective stimulator of IL-6 expression in human microglia, LPS elicited a higher expression of the cytokine. Indeed, bands for IL-6 appeared saturated (Fig 2B) and showed no change in intensity with increased number of PCR cycles (data not shown). Saturation with LPS would prevent a quantitative comparison between PAF and LPS as activating stimuli for microglial expression of IL-6 (Fig 2C). An interesting observation was that SKF96365 partially inhibited the LPS-induced expression of IL-6 (Fig 2C). Although LPS has been reported to act in a Ca^2+^-independent manner on macrophages [19], several studies have found the bacterial compound evokes changes in [Ca^2+^]i in microglia/macrophages [1,5,16,32] suggesting possible involvement of SOC in LPS induction of cytokines.

The present results may have relevance to roles of IL-6 in aging. Several studies have provided evidence for age-dependent increases in levels of IL-6 in rodent brain [reviewed in [10]]. For example, one finding was that brains from older mice showed considerable elevations in expression and production of IL-6 compared with brains from younger animals [33]. This result was correlated with microglial production of the cytokine [33]. It will be of interest to determine if PAF-stimulated adult human microglia are more potent producers of IL-6 compared with fetal human cells.

## List of abbreviations

PAF: platelet-activating factor; SOC: store-operated channels; IL-6; interleukin-6; PSS: physiological saline solution; PBS: phosphate-buffered saline; [Ca2+]i: intracellular calcium; DMEM: Dulbecco's modified Eagle's medium

## Competing interests

The author(s) declare that they have no competing interests.

## Authors' contributions

PS and HBC contributed equally to calcium imaging, RT-PCR and ELISA experiments. HBC also carried out isolation of microglia. SC participated in the design of experiments and reviewed and edited the manuscript. JGM designed and supervised all experiments, interpreted the data and finalized the manuscript. All authors read and approved the final manuscript.
